# Codesigning a Nursing Leadership Program to Transform Value and Delivery Systems for Fundamental Care

**DOI:** 10.1155/2023/1318377

**Published:** 2023-08-31

**Authors:** Rebecca Feo, Susanne Pearce, Alison Kitson, Tiffany Conroy

**Affiliations:** College of Nursing and Health Sciences, Flinders University, Sturt Road Beford Park, Adelaide, SA 5042, Australia

## Abstract

**Aim:**

The aim of this article is to report the process and outcomes of codesigning a nursing leadership program for fundamental care. The leadership program is designed to empower nursing leaders, across research, education, clinical practice, and policy, to challenge and change how fundamental care is valued, prioritised, and actioned within health and care systems.

**Background:**

Deficits in fundamental care represent an intractable problem adversely impacting care recipients, care providers, and health and care systems globally. These deficits stem from the minimal value placed on fundamental care and its subsequent invisibility across research, education, clinical practice, and policy. Sustainable systems change requires effective nursing leadership; however, existing nursing leadership programs tend to address only one area of health and care systems, typically clinical practice, and do not focus specifically on fundamental care.

**Methods:**

The Fundamentals of Care Leadership Program was codesigned with current and emerging nursing leaders using a participatory action research approach. The collaborative codesign process involved two stages: (1) idea generation and preliminary program development via Nominal Group Technique (*n* = 60 participants from 11 countries) and (2) refinement and trialling of program content and process via a three-day workshop (*n* = 19 participants from 9 countries).

**Results:**

Participants prioritised a program that provided clear understanding of the concept of fundamental care, enabled the development of influencing and negotiating skills to advocate for this care, and offered resources on knowledge translation, implementation, and measurement strategies. Participants also wanted allotted time to design research and quality improvement projects that would allow them to transfer the skills learned to the real-world issues occurring within their respective organisations.

**Conclusions:**

The codesign process, embedded within a participatory action approach, enabled the development of a Fundamentals of Care Leadership Program that is shaped by, and meets the needs of, current and emerging nursing leaders. The leadership program will enable nursing leaders to challenge value systems on fundamental care and to champion this care across research, education, clinical practice, and policy, working towards enhanced fundamental care outcomes and experiences.

## 1. Introduction

### 1.1. Background

Fundamental care is often considered the foundation of nursing practice [1], underpinning seminal nursing theories as well as frameworks and guidelines for care delivery [2–4]. Fundamental care is defined as nurse actions that respect and focus on a person's essential needs to ensure their physical and psychosocial wellbeing [5]. These needs are met through a trusting, therapeutic relationship between care providers and care recipients, whilst taking into consideration the context in which care is taking place [5]. Despite the centrality of fundamental care to nursing practice and care outcomes, internationally, care recipients report negative experiences of this care, including missed, infrequent, or poor-quality mouth care, toileting, bathing, mobility, information provision, education, and psychosocial support [6–11]. In turn, care recipients experience numerous adverse outcomes when fundamental care is poorly delivered, including pressure injuries, falls, new infections, readmissions, and death [8, 12–17].

There are several complex reasons for the global deficits in fundamental care. They include the predominance of biomedical approaches to care, the prioritisation of productivity and efficiency within health and care systems, and workforce issues (e.g., high workloads and inadequate nurse-to-patient ratios) [18–22]. As a result of these pressures, fundamental care, particularly psychosocial and relational aspects, is afforded minimal priority, rendering it invisible and devalued across health and care systems [19]. For a decade, there have been calls to address the invisibility of fundamental care by empowering nursing leaders to champion change and affect a cultural shift in the way in which this care is valued and enacted at micro, meso, and macrolevels of health and care systems [23–26]. Commensurate with these calls is a growing body of research on leadership for fundamental care, albeit focused primarily on how this leadership relates to the delivery of fundamental care within clinical practice [26]. This research has shown that care recipients, nurses, and nurse managers all view appropriate and effective nursing leadership as central for facilitating high-quality fundamental care delivery [27, 28].

The importance of nursing leadership is not a new concept; more than 20 years ago, researchers argued that effective leadership was critical for positively influencing nursing practice and policy [29, 30]. In turn, there are numerous leadership programs designed to enhance nurses' leadership capabilities and to influence change [31, 32]. More recently, there is a growing body of work on how different leadership styles, particularly relational styles such as transformational leadership, can positively influence nursing practice, care-recipient outcomes, and nurses' job satisfaction [33–35]. Nursing leadership was emphasised once again during the global COVID-19 pandemic, with a focus on how effective leadership during times of crises can maintain quality standards and nurses' emotional and mental well-being, in both academic and clinical practice settings [36–38].

Despite these advances, knowledge and action around leadership specifically for fundamental care are lacking. Fundamental care remains a relatively under-researched area of nursing leadership [39], and there is limited guidance to support nursing leaders across all areas and at all levels of health and care systems to champion change in the way in which this care is valued, prioritised, and actioned [26]. Furthermore, the sustained deficits in fundamental care globally demonstrate that existing leadership programs, whilst undoubtedly successful in influencing change in some areas [40], are not necessarily supporting leaders to affect systems change in ways that translate to improved fundamental care outcomes and experiences. This is likely for two reasons. First, many existing leadership programs focus on only one aspect of health and care systems, most commonly clinical practice [41, 42], without an accompanying focus on leadership within research, education, or policy. Second, programs typically focus on general leadership capabilities rather than specifically on fundamental care and the individual system and policy level factors that influence its perceived value and subsequent prioritisation within health and care systems.

To achieve sustained system-level impact for fundamental care, leadership programs must influence all facets of healthcare: research, education, clinical practice, and policy. Continuing to operate in siloes will only perpetuate poorly integrated health and care systems, where nursing education does not readily translate to real-life clinical practice; practising nurses find it difficult to see the relevance of research evidence, and policy initiatives do not reflect the realities of everyday clinical practice [43–45]. In addition to focusing on all areas of health and care systems, leadership programs must focus specifically on fundamental care rather than general leadership capabilities. The nursing education literature demonstrates that when fundamental care is not explicit nor reinforced within nursing curricula, it is rendered invisible and devalued [46, 47]. Implicitly embedding fundamental care within a general leadership program is similarly insufficient. Health and care systems already fail to value the role of fundamental care in enhancing care outcomes and experiences [19]; leadership programs that do not explicitly emphasise fundamental care only reinforce this devaluing. The need for targeted nursing leadership programs is also supported by existing literature, which has argued the importance of designing and implementing tailored programs that address the different facets of nursing leadership [48, 49].

To support nursing leaders in changing deeply entrenched organisational cultures and value systems on fundamental care, they must have the knowledge and skills to advocate for this care in a politically informed way and to foster sustainable change [50]. The International Learning Collaborative (ILC) is supporting nursing leaders to develop the skills required to affect real systems change for fundamental care. The ILC is a member-based international network aiming to transform how fundamental care is delivered, taught, and researched globally. It has been a long-held vision of the ILC to grow a critical mass of leaders globally who are courageous and skilled to champion and lead change. To achieve this vision, the ILC has engaged with current and emerging nursing leaders worldwide to codesign the Fundamentals of Care Leadership Program. The program aims to enhance the capacity of current and emerging nursing leaders within research, education, clinical practice, and policy to advocate for and affect systems change around fundamental care, thus ensuring the delivery of high-quality fundamental care within any health or care environment. This article outlines the collaborative codesign process undertaken to develop the leadership program and considers how this program might be sustained into the future.

## 2. Methods

### 2.1. Design

The development of the Fundamentals of Care Leadership Program was underpinned by participatory action research (PAR) [51, 52]. PAR requires active involvement of key stakeholders—those with a vested interest in or potential to benefit from the research findings—in the research process, with the aim of generating societal and system-level change [52, 53]. In the present study, the first step of the PAR process involved several discussions amongst the ILC's governing body (its steering committee) regarding how best to meet the challenge of shifting prevailing organisational cultures and value systems on fundamental care. At the time this study was conducted, the ILC's steering committee comprised of 13 nursing leaders from eight countries, all with relevant leadership expertise across research, education, clinical practice, and/or policy. Through these discussions, and based on their own leadership experiences, the steering committee identified the need for a leadership program specifically for fundamental care that would equip nursing leaders with the knowledge and skills to effect change at a systems-level and which would meet the needs of a global nursing audience.

**Figure 1:**
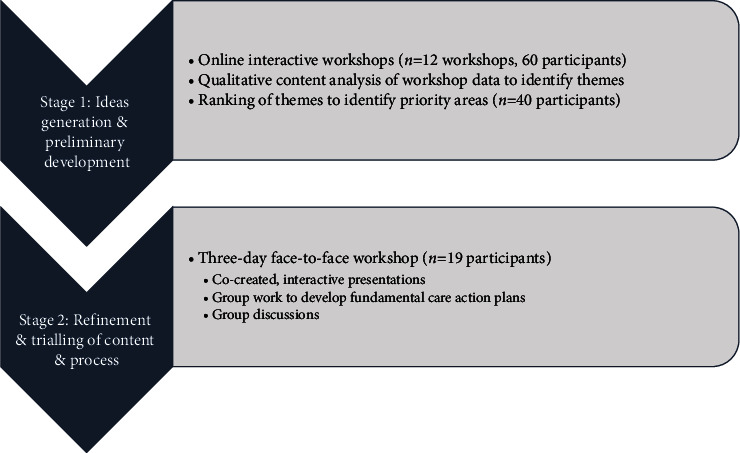
Fundamentals of care leadership program codesign development process.

Aligning with the PAR principles of collaboration and participation [53], the proposed leadership program was then codesigned with an international cohort of nursing leaders via an iterative, two-stage process (see also Figure 1).

- Stage 1: Idea generation and preliminary program development via modified nominal group technique
- Stage 2: Refinement and trialling of program content and process via a three-day workshop

The codesign process drew on the expertise and experience of both current and emerging nursing leaders within and across healthcare research, education, clinical practice, and policy. Current nursing leaders were defined as individuals who successfully influence others to achieve a common goal and who are often employed in managerial or executive positions within their organisations [54]. Emerging nursing leaders were defined as individuals who are developing the skills to influence others but who might not yet occupy designated leadership positions.

### 2.2. Stage 1: Idea Generation and Preliminary Program Development

Aligning with the PAR approach, the first stage of the codesign process involved working with current and emerging nursing leaders to identify their priorities and preferences for a leadership program on fundamental care. This was achieved via nominal group technique (NGT) [55]. NGT is a structured consensus-generating approach that aims to develop ideas and solutions to a problem and facilitate agreement on the relative importance of the proposed ideas/solutions [55]. This study used a modified NGT approach [56, 57], involving online interactive workshops to enable idea generation, sharing, and clarification, follwed by an online ranking process to determine which ideas participants prioritised.

#### 2.2.1. Online Interactive Workshops

The workshops were conducted in June 2021 as part of the ILC's annual conference. The aim of the workshops was to identify the leadership program's goals, parameters, and deliverables.

*(1) Setting and Participants*. Workshops took place online via Zoom breakout rooms. Sixty participants from 11 countries (Australia, Canada, Denmark, Italy, Japan, New Zealand, Sweden, the Netherlands, Norway, the UK, and the US) took part. Participants self-selected to participate upon registration to the ILC conference.

*(2) Data Collection*. Four workshops were run simultaneously, each focusing on a different area: research, education, clinical practice, and policy. Participants nominated the workshop they preferred to participate in. Workshops were repeated a further two times, enabling participants to take part in workshops on different topics (*n* = 12 workshops total). Workshops were guided by a template with open-ended prompts (see Table 1). The template was developed by the first author with the expertise of the ILC's steering committee. Each workshop ran for 45 minutes and was managed by a facilitator, with a maximum of 15 participants. Workshops were audio and video recorded.

*(3) Data Analysis*. Following transcription of workshop recordings, the data were analysed using qualitative content analysis (QCA) [58, 59]. QCA allows for a focus on the frequency of patterns within the data [59], thus aligning with the NGT aim of consensus-generation. Following initial reading and familiarisation of the written transcripts, the data were coded inductively and deductively. The second author coded the data to the workshop questions and identified additional codes that did not align with these questions. Data from the different workshops, research, education, clinical practice, and policy, were considered as one dataset rather than separate as the initial familiarisation showed that there were no substantial differences in the content generated between the different workshops. Codes were refined via discussion with all authors, with the first and second authors then categorising the codes, based on similarity, into themes. The themes were further refined following discussion with all authors, nursing academics at Flinders University, and the ILC's steering committee.

#### 2.2.2. Online Ranking Process

The ranking process involved all workshop participants being invited to rate their preferences, from a list of options derived from the QCA, in relation to the leadership program content and outcomes.

*(1) Setting and Participants*. Participants were sent a link to an online Qualtrics survey in February 2022. Of the participants who took part in the workshops (*n* = 60), forty responded to the survey (response rate = 67%).

*(2) Data Collection*. The online survey consisted of four sections:

1. Program content
2. Program activities
3. Program outcomes/outputs
4. Draft program

**Figure 2:**
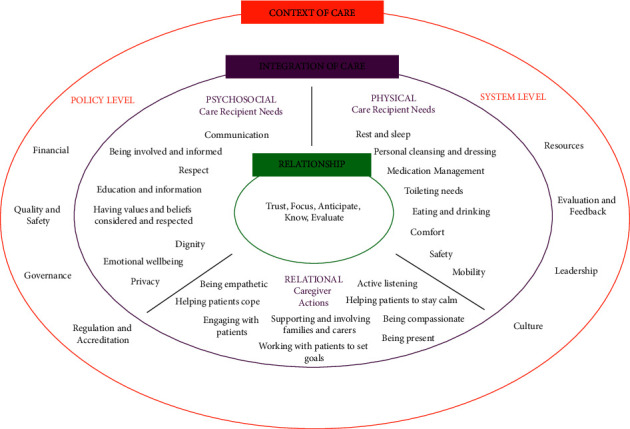
The Fundamentals of Care Framework. Image was obtained, with permission, from https://ilccare.org/the-framework/ and the content within the image was derived from [5].

Sections 1–3 each had predetermined responses, derived from the analysis of the workshop data. Participants were asked to rate their top five responses in order of preference, with 1 being the most preferred. The ranking of five priority ideas is common in NGT [55].  Section 4 provided a draft of the proposed leadership program, developed based on the ideas generated in the workshops. Participants were asked to provide feedback on this draft via a series of open-ended questions. The draft program comprised four parts:

- Part 1: An overview of the Fundamentals of Care Framework [25]. The Fundamentals of Care Framework is a conceptual framework, developed by the ILC, that outlines what high-quality fundamental care should look like in clinical practice. The Fundamentals of Care Framework outlines three core dimensions for high-quality fundamental care delivery: (1) developing trusting relationships with care recipients and carers; (2) integrating and addressing care recipients' physical, psychosocial, and relational needs; and (3) being aware of how the care context can influence care delivery (see Figure 2).
- Part 2: Group discussion of self- and context-assessments, completed by participants in their own time prior to the Stage 1 workshops. These assessments determined participants' readiness for leading transformation as well as their organisations' readiness for change.
- Part 3: Working with a mentor to develop a fundamental care action plan around an identified area of need and presenting the plan to all participants for feedback.
- Part 4: A one-day debrief 12 months after program completion, enabling participants to provide an update on their action plans and share their experiences with the next cohort.

*(3) Data Analysis*. The top five responses for Sections 1–3 were collated. The open-ended responses in Section 4 were analysed via QCA.

#### 2.2.3. Ethical Considerations

Ethical approval was received from Flinders University Human Research Ethics Committee (approval number: HEL1858). Participants were informed upon registration that the workshops would be video and audio recorded and that their participation in the workshop implied their consent for the recordings to be used in the research. The ranking survey was anonymous.

### 2.3. Stage 2: Refining and Trialling of Program Content and Process

The last stage of the codesign process used the ideas prioritised by the participants in Stage 1 to refine and trial the content, structure, and process of the leadership program. The result would be a structured leadership program for piloting in 2023. Stage 2 consisted of a three-day, face-to-face, codesign workshop led by the third and fourth authors.

#### 2.3.1. Setting and Participants

The workshop took place in June 2022 at Saïd Business School, Oxford University, UK. Current and emerging nursing leaders were identified from the ILC's steering committee, organisational members, and international networks and were invited to take part. Participants were selected to ensure a breadth of experience and geographical representation. Nineteen participants from nine countries (Australia, Canada, Denmark, Iceland, New Zealand, Norway, Sweden, the UK, and the US) took part.

#### 2.3.2. Data Collection

The workshop consisted of interactive presentations followed by the development of fundamental care action plans. These were interspersed with group discussion where participants provided real-time feedback on and subsequently refined the proposed program content.

*(1) Cocreated Interactive Presentations*. Prior to the workshop, participants were allocated to one of three groups and tasked with cocreating a presentation on a specified topic that could form a part of the 2023 pilot. The topics, derived from the prioritisation process in Stage 1, were as follows.

- Overview of fundamental care and the Fundamentals of Care Framework
- Influencing change in fundamental care now and into the future
- Knowledge translation and implementation strategies

Groups delivered their presentations to the wider group for refinement. This refinement took place during the presentations themselves, with participants offering suggestions for improvement.

*(2) Fundamental Care Action Plans*. Participants self-selected into groups to develop a fundamental care action plan, a tailored approach to addressing value systems and leading change at multiple levels within their respective organisations. The aim of including action plan development in the codesign process was to test how this development might work practically within the leadership program. Each group had a fellow participant who acted as a mentor to assist in plan development. Mentors were self-nominated or identified by groups based on relevant expertise. The groups were tasked with delivering a 10-minute presentation on their action plan to the wider group and reviewing the plan based on group feedback.

*(3) Group Discussions*. Time was allotted across the three days for group discussion on the evolving leadership program content, including participants' recommendations for the 2023 pilot. The third and fourth authors made notes of these discussions.

#### 2.3.3. Data Analysis

The notes from group discussions were consolidated by the third author to generate the next iteration of the leadership program.

#### 2.3.4. Ethical Considerations

Ethical approval was granted by Flinders University Human Research Ethics Committee (approval number: HEL1858). Participants were informed that notes would be taken of group discussions, which would be used for program development and research.

## 3. Results

### 3.1. Stage 1: Idea Generation and Preliminary Program Development

#### 3.1.1. Online Interactive Workshops

Four main themes were identified from the workshop data, as illustrated in Table 2. The first theme, “advocacy and lobbying,” demonstrated that participants wanted a leadership program that provided them with the tools to advocate and speak up for fundamental care across health and care systems and with their interdisciplinary colleagues. The second theme, “understanding fundamental care,” demonstrated that participants wanted a program that would strengthen their conceptual understanding of fundamental care, including the use of appropriate terminology. The third theme, “measurement and implementation,” described participants' preference for a program that would equip them with the skills to identify and use appropriate implementation and measurement strategies, enabling them to effect real change within their respective organisations. In the fourth theme, “providing a supportive network,” participants emphasised the importance of the leadership program providing a forum where program participants can motivate one another and build global connections, enabling collaborative problem-solving.

#### 3.1.2. Online Ranking Process

The results from the ranking process are presented in Table 3. Participants prioritised program content and outputs that focused on understanding the concept of fundamental care, implementation strategies, advocating for fundamental care, designing quality improvement or research projects relevant to their organisation, self and organisational readiness assessments, and fundamental care measurement tools. The importance of the leadership program providing a supportive network was not prioritised.

Participants' responses to the draft leadership program were positive, describing it as well-structured, relevant, comprehensive, and innovative. Participants liked the 12-month follow-up, the use of mentors, and the practical focus, exemplified by the action plans. Participants also identified potential gaps in the program, offering the following solutions: (1) providing participants with a summary of fundamental care and the Fundamentals of Care Framework prior to the program to assist with self- and context-assessments, (2) including a stronger focus on knowledge translation and implementation in both the prereading and program content, and (3) incorporating a focus on sustainability into the action plan development.

### 3.2. Stage 2: Refinement and Trialling of Program Content and Process

Table 4 provides an outline of the revised Fundamentals of Care Leadership Program. Based on participants' real-time feedback, the leadership program was refined to include additional prereading and video resources on the Fundamentals of Care Framework and knowledge translation. To assist with action plan development, the program will also include additional resources and content on project planning, program logic, and mentoring. Seven fundamental care action plans were successfully developed during the three-day workshop, demonstrating the feasibility of including this process within the leadership program. The plans ranged in content from establishing regional ILC networks in America and Sweden to measuring the inter-related dimensions of the Fundamentals of Care Framework; generating an innovative, strategic approach to introducing fundamental care into preregistration curricula; and implementing the Fundamentals of Care Framework into clinical practice to guide care delivery. Each group is being provided structured, virtual mentoring over 12 months, with evaluation to occur thereafter.

## 4. Discussion

Improving fundamental care requires nursing leaders to challenge and change how this care is valued within health and care systems [19, 25]. The Fundamentals of Care Leadership Program aims to equip current and emerging nursing leaders with the skills and knowledge to make this change a reality. Codesigning the program through a participatory action research approach has generated considerable buy-in for fundamental care and its leadership globally, as illustrated by the number of participants across both stages and the number of action plans developed. The participatory action codesign approach also provided a platform for participants, who were at all levels and areas of health and care systems, to see the value in and advocate for fundamental care. The leadership program will be available to all current and emerging nursing leaders, regardless of whether they are ILC members, and will be hosted face-to-face in a different country each year, thus enhancing accessibility.

Whilst our leadership program is designed to challenge value systems, making sure the program gains traction and is sustained will be a system's challenge in itself. Numerous leadership programs have shown initial promise yet failed to sustain long-term impact. To avoid our program following the same path, we must ensure it achieves its vision and, in doing so, secures a place in the broader health and care landscape. In this discussion, we consider what we might learn from other healthcare movements, namely, quality and safety and evidence-based practice, as well as the few larger-scale fundamental care initiatives that are gaining momentum, at least at national levels. Our aim is not to provide an exhaustive overview of these agendas; however, learning from their relative success will help to identify some of the policy and systems levers that we must consider in ensuring the Fundamentals of Care Leadership Program is successful in the long term.

The quality and safety movement, which focuses primarily on keeping care recipients safe from preventable harm [60], has gained considerable traction worldwide. It forms a part of the World Health Organisation's agenda [61] and has spawned several agencies, commissions, policies, and journals globally. The quality and safety movement has likely gained traction for three reasons: (1) it has primarily been medically driven [60]; (2) it aligns with the risk-averse culture of most health and care systems [19]; and (3) it has a strong economic driver (i.e., safety incidents cost money) [62, 63]. Like quality and safety, the evidence-based practice movement has gained traction in the last few decades. Although an evidence-practice gap still exists in healthcare [64], evidence-based practice is nonetheless seen to hold value. This is likely because it also relates to discourses around harm and risk, that is, failing to practise based on the latest evidence means possibly providing care that is ineffective, unnecessary, and unsafe. Fundamental care cannot necessarily or immediately leverage some of the drivers that have underpinned quality and safety and evidence-based practice; for instance, the economic case for relationship-based, integrated fundamental care, as espoused in the Fundamentals of Care Framework, remains unclear [65]. Nonetheless, there are lessons to be learnt from these agendas in terms of gaining traction for, and ensuring sustainability of, the Fundamentals of Care Leadership Program.

First, we must consider whether and how to align fundamental care initiatives, including the leadership program, with existing healthcare agendas. We know that failures in fundamental care compromise care quality and safety. Ensuring the safety of care recipients is also a key aspect of fundamental care delivery, as exemplified by the Fundamentals of Care Framework (see Figure 2 and [66]). Perhaps the goal for nursing leaders, then, is to incorporate fundamental care into quality and safety policies, agendas, and standards. Aligning with the quality and safety movement might also strengthen the economic case for fundamental care, demonstrating the monetary cost to health and care systems for failing to deliver this care to a consistently high standard. However, nursing leaders must navigate carefully; aligning with quality and safety can potentially reinforce, rather than challenge, prevailing value systems focused primarily on risk aversion [19]. Our leadership program must therefore empower nursing leaders to advocate for fundamental care as central rather than peripheral to existing care agendas [50]. The work of nursing leaders at Sinai Health System provides an example of how this can be achieved [67], that is, how we can leverage and even shape existing health and care drivers and values.

The Sinai Health System team, through collaborative partnership between leaders and clinicians in academia and clinical practice, has developed an evidence-informed Science of Care Framework. This framework situates fundamental care as the nexus point that intersects caring with safety, symptom, implementation, improvement, and innovation sciences, to provide holistic guidance for care delivery [67]. Efforts such as this align with but also expand current understandings of quality and safety, working to reframe what health and care systems value. By placing high-quality fundamental care as the goal of care delivery, the Science of Care Framework positions quality and safety as a mechanism to support fundamental care rather than as the driving force of care delivery itself. This also aligns with calls for the quality and safety agenda to evolve by shifting away from a focus on risk and harm, towards codesigned safety measures that encapsulate what matters most to those receiving care, including relational aspects [67]. Whilst the Science of Care Framework is yet to be tested in clinical practice, it is an example of how we can work with, rather than against, existing healthcare agendas to challenge value systems and advance fundamental care. By equipping nursing leaders with the courage, knowledge, and skills to engage in partnership and advocate for the centrality of fundamental care, the Fundamentals of Care Leadership Program can meet its intended vision and ensure its continued presence in the health and care landscape.

A second lesson to be learnt from existing healthcare movements is that gaining traction is only one piece of the puzzle. Even the quality and safety agenda has more work to do in terms of ensuring long-term success and widespread adoption of safety interventions [60]. Hence, changing value systems around fundamental care, whilst a crucial first step, will not automatically and inevitably translate into improved care. Our leadership program must also equip nursing leaders with the requisite skills to implement and evaluate new ways of thinking, practising, and collaborating across research, education, clinical practice, and policy. There are local, small-scale efforts to improve fundamental care in these areas [23, 68]; however, large-scale efforts are rare [67, 69, 70]. The Fundamentals of Care Leadership Program must provide current and emerging nursing leaders with the skills to scale-up local initiatives, thus enabling change in the valuing, prioritisation, and actioning of fundamental care at micro, meso, and macrolevels. By becoming courageous and skilled leaders at all levels and areas of health and care systems, nurses will substantially shift organisational and system values for fundamental care, ultimately enhancing how this care is delivered.

## 5. Conclusions

Nurses are present in all areas and levels of health and care systems and make up the largest healthcare profession globally; their potential to influence culture, value systems, and care delivery must be fostered. A leadership program for fundamental care that, at its core, emphasises the value of this care will enable nursing leaders to positively influence how fundamental care is perceived and actioned within and across health and care systems. Using a participatory action approach to codesign, we were able to draw on the collective knowledge and active involvement of current and emerging nursing leaders globally, ensuring the development of a leadership program for fundamental care that is relevant and targeted to its intended audience and proposed aim. The resultant Fundamentals of Care Leadership Program can equip current and emerging nursing leaders across research, education, clinical practice, and policy with the requisite skills and knowledge to challenge value systems on fundamental care and to champion and sustain much-needed change, thus working towards improved care delivery and outcomes. Our goal moving forward is to assess the impact of the program and ensure its continued sustainability.

## Figures and Tables

**Table 1:** Workshop template.

*Role of facilitators*
Your role is to elicit the required information from workshop participants. Use the below prompts to encourage group discussion. If you are facilitating multiple groups on the same topic, feel free to summarise the results from the previous group and encourage participants to add their thoughts

*Prompts*
(1) What should the leadership program deliver in terms of attitudes, behaviours, skills, and competencies for nursing leaders?
(2) How would we know that a leadership program for nursing leaders had achieved its objectives? What could we observe or measure to determine this?
(3) Which nursing leaders should the leadership program be aimed at?
(4) What would be the unique selling point of a leadership program focused on fundamental care for nursing leaders?
(5) How can nursing leaders identify and tap into opportunities for enhancing and leading fundamental care research/education/clinical practice/policy?
(6) What else do nursing leaders need to know to lead change around fundamental care research/education/clinical practice/policy?

**Table 2:** Main themes identified in Stage 1 with examples of illustrative quotes.

Theme	Examples of illustrative quotes from participants
Advocacy and lobbying	The [leadership program] needs to develop brave and courageous leaders who are not afraid to speak up, irrespective of the consequences
	[Fundamental care] is important at all levels of healthcare, not just nursing. Leaders need to have the ability to influence and gain support from other leaders outside of nursing
	Nurse leaders… they're not trained in or skilled in advocating for nursing. They are trained in leadership, they take masters' degrees in public administration and public leadership and so forth, [but] they're not equipped to advocate or speak up for nursing, so a leadership program would have to equip them [with] that

Understanding fundamental care	Leaders, irrespective of their health profession, need to understand what fundamental care means
	Creating a common, accessible, and consistent language about fundamental care

Measurement and implementation strategies	It is easy to believe that fundamental care is important but more difficult to implement into practice. The leadership program should assist people in how to implement changes in fundamental care in their setting
	Quality indicators in healthcare are often centred around process-type measures or misses and near misses, but they really don't address the core of the Fundamentals of Care Framework, which is that relationship… it would be nice to learn how to measure this

Providing a supportive network	Developing a network of fellow nurses, researchers, and leaders would be very motivating. It helps people to connect internationally and discuss challenges
	The program could provide a platform for worldwide fundamentals of care initiatives to inspire others, connect, and build stronger networks

**Table 3:** Results from the Stage 1 NGT ranking process, showing the top five rated responses.

	(%)
*Section 1: Program content*	
(1) Implementation strategies (in research, education, clinical practice, and policy)	16.36
(2) Understanding what fundamental care is and how to deliver it using the Fundamentals of Care Framework	14.49
(3) Influencing, lobbying, advocacy, empowering, and challenging value systems	11.21
(4) Measuring fundamental care	9.81
(5) Partnership building/collaboration (with care recipients, healthcare professionals, executives)	7.48
(6) Leadership styles, attributes, and skills	7.48

*Section 2: Program activities*	
(1) Participants choose a quality improvement or research project	24.19
(2) A self-assessment for fundamental care readiness (knowing what leader they are now and what they need to be a leader for fundamental care in the future)	22.58
(3) An organisational assessment for fundamental care readiness	16.94
(4) Development of a fundamental care measurement tool	12.90
(5) A quality improvement project that includes three phases: Phase 1: Diagnose (issue and evidence), Phase 2: Plan and prioritise (recommendations, policy impact, responsibility), and Phase 3: Implement and evaluate	11.29

*Section 3: Program outcomes/outputs*	
(1) Participants are better equipped to implement fundamental care in their workplace	18.09
(2) Participants have tools to advocate for fundamental care and are more confident to speak out for fundamental care	15.08
(3) Participants more strongly advocate for the nursing profession	14.57
(4) Participants have a better understanding of their own and the organisational barriers and enablers to fundamental care	12.56
(5) Participants and organisations have access to long-term support to implement and monitor fundamental care	10.55

**Table 4:** An outline of the Fundamentals of Care Leadership Program to be piloted in 2023.

Prereading	Key readings and video resources on:
	(i) Why we need to focus on fundamental care
	(ii) The development of the Fundamentals of Care Framework
	(iii) Mentoring
	(iv) Leadership and influencing
	(v) Knowledge Translation and implementation

Day 1	Presentations and group work:
	(i) Session 1: Making the case–Why fundamental care matters
	(ii) Session 2: What is fundamental care–The history of the Fundamentals of Care Framework
	(iii) Session 3: Knowledge translation–How to get fundamental care into research, education, clinical practice, and policy

Day 2	Presentations and group work:
	(i) Session 4: Project planning and program logic, including preliminary development of fundamental care action plans
	(ii) Session 5: Leadership and influencing
	(iii) Session 6: Mentoring–What it is, why it matters, and how you do it

Day 3	Receiving feedback on and finalising fundamental care action plans

## Data Availability

The workshop data from Stage 1 used to support the findings of this study have not been made available to protect participant confidentiality and anonymity (the workshops were video and audio recorded, which means the participants are easily identifiable). The transcripts of the workshops and the data from the Stage 1 ranking process are restricted by the Flinders University HREC to protect participant confidentiality. Data are available from the first author for researchers who meet the criteria for access to confidential data.
